# Incidence and Risk Factors for Superficial and Deep Vein Thrombosis in Post-Craniotomy/Craniectomy Neurosurgical Patients

**DOI:** 10.7759/cureus.32476

**Published:** 2022-12-13

**Authors:** Bhavika Gupta, Mohammed B Uddin, Kyle Rei, Christopher Andraos, Vedhika Reddy, James Brazdzionis, Samir Kashyap, Javed Siddiqi

**Affiliations:** 1 Neurosurgery, Arrowhead Regional Medical Center, Colton, USA; 2 Neurosurgery, California University of Science and Medicine, Colton, USA; 3 Neurosurgery, Riverside University Health System Medical Center, Moreno Valley, USA; 4 Neurosurgery, Desert Regional Medical Center, Palm Springs, USA

**Keywords:** venous thromboembolism prophylaxis, deep vein thrombosis, venous thromboembolism, neurosurgery, craniectomy, craniotomy, brain tumor, neurotrauma, superficial vein thrombosis, post-neurosurgery complication

## Abstract

Background

Venous thromboembolism (VTE) is quite common among post-operative neurosurgical patients. This study aims to identify the incidence of deep vein thrombosis (DVT) and superficial vein thrombosis (SVT) among post-craniotomy/craniectomy patients and further evaluate established hypercoagulability risk factors such as trauma, tumors, and surgery.

Methodology

This single-center retrospective study investigated 197 patients who underwent a craniotomy/craniectomy. The incidences of DVT and SVT were compared, along with laterality and peripherally inserted central catheter (PICC) line involvement. A multivariate logistic regression analysis was conducted to identify risk factors for post-craniotomy/craniectomy VTE. This model included variables such as age, post-operative days before anticoagulant administration, female sex, indications for surgery such as tumor and trauma, presence of a PICC line, and anticoagulant administration.

Results

Among the 197 post-craniotomy/craniectomy patients (39.6% female; mean age 53.8±15.7 years), the incidences of DVT, SVT, and VTE were 4.6%, 9.6%, and 12.2%, respectively. The multivariate logistic regression analysis found that increasing the number of days between surgery and administration of anticoagulants significantly increased the risk of VTE incidence (odds ratio 1.183, 95% CI 1.068-1.311, p = 0.001).

Conclusions

Contrary to existing evidence, this study did not find trauma or the presence of tumors to be risk factors for VTE. Future prospective studies should assess VTE risk assessment models such as “3 Bucket” or “Caprini” to develop universal guidelines for administering anticoagulant therapy to post-craniotomy/craniectomy patients that consider the timing of post-operative therapy initiation.

## Introduction

Venous thromboembolism (VTE), which includes deep vein thrombosis (DVT), superficial vein thrombosis (SVT), and pulmonary embolism (PE), can be life-threatening and affects approximately 10 million Americans annually [1]. DVT occurs when a blood clot forms in the deep veins of the pelvis or legs or less frequently in the arms [2]. Peripheral venous thrombosis is among the most common post-operative complications in neurosurgical patients [3]. In understanding why neurosurgical patients are at high risk of developing a VTE, Virchow’s triad offers a useful framework: stasis, endothelial injury, and hypercoagulable state. Venous stasis occurs postoperatively due to decreased mobility at the time of recovery, and due to the severity of many neurosurgical procedures, recovery and ambulation are frequently protracted [4]. Endothelial damage happens due to tissue injury and focal ischemia that is secondary to traumatic brain injury (TBI), intracranial hemorrhage, and neo-angiogenesis caused by brain tumors [4,5]. Lastly, a hypercoagulable state occurs secondary to an inflammatory environment created by the stress of surgery and the presence of a space-occupying lesion, especially when it is malignant [6]. Among neurosurgical patients, the incidence of DVT ranges from 12.0% to 26.1% [1-3]. Such an incidence can be attributed to trauma, tumors, and surgery, which have been identified as important risk factors for hypercoagulability in critically ill patients [2,3].

Traumatic brain injury

TBI is a risk factor for hypercoagulability because shearing forces and localized ischemia secondary to brain injury can disrupt vessel integrity and expose the brain parenchyma to plasma. The brain parenchyma is rich in thromboplastin, and its secretion increases significantly in the case of an injury or stress. Consequently, when the brain parenchyma is exposed to plasma, thromboplastin or procoagulant tissue factor combines with factor VII of the extrinsic clotting cascade and activates the coagulation pathway [6,7].

Tumors

It is believed that patients with brain tumors are at increased risk of hypercoagulability because brain tumor microenvironments increase the secretion of procoagulants such as tissue factor and inhibit anticoagulants such as plasmin. This leads to an imbalance in the clotting cascade, which leads to hypercoagulability [8]. Additionally, both primary brain malignancy and metastases to the brain are associated with the breakdown of the blood-brain barrier, which, similar to the mechanism described for TBI, activates the coagulation pathway [9]. Consequently, approximately 20-30% of patients with brain tumors will develop VTE [10,11]. Among both primary and secondary brain tumors, glioblastoma is associated with the highest rate of VTE incidence due to increased expression of tissue factor and podoplanin. Tissue factor is directly involved in the coagulation cascade, and adherence of podoplanin to C-type lectin receptor-2 is hypothesized to induce clotting [12].

Surgery

Undergoing craniotomy/craniectomy is a risk factor for hypercoagulability regardless of indication. Tactile stimulation of the brain parenchyma during surgery increases the release of tissue factor, peaking at the third hour of surgery [13]. Additionally, due to the complexity of many neurosurgical procedures, operative times commonly exceed three hours, during which the patient is sedentary with increased venous stasis [13].

Peripherally inserted central catheter (PICC) line 

Lastly, PICC lines are also a risk factor for hypercoagulability. Nearly 61.9% of patients with PICC lines develop SVT or occasionally DVT if anticoagulation therapy is not administered compared to only 22.9% of patients who receive prophylactic anticoagulation therapy [14]. It is hypothesized that PICC lines increase the risk for VTE because when inserted into small peripheral veins they reduce blood flow and promote venous stasis. Additionally, the placement of PICC lines cause direct endothelial injury [15].

This study aimed to identify the incidence and risk factors associated with DVT and SVT among post-craniotomy/craniectomy neurosurgical patients at Arrowhead Regional Medical Center.

## Materials and methods

This single-center retrospective study was performed at Arrowhead Regional Medical Center, a tertiary care trauma and stroke center in Southern California. In this study, patients over the age of 18 who underwent a craniotomy/craniectomy between July 2017 and June 2022 were included. However, patients with a history of coagulopathy and those who were admitted to the neurocritical care unit less than 48 hours after the surgery were excluded. Baseline data were collected through a manual chart review of all patients and included information on their age, sex, indication for surgery, laterality of DVT/SVT, presence of PICC line, and status of anticoagulant administration. 

Statistical analyses were performed using SPSS version 28.0.1.0 (142). Summary statistics for quantitative data have been reported using mean ± standard deviation (SD) and nominal data as percentages. Qualitative variables were analyzed using the Chi-square (ꭓ^2^) test, and quantitative variables were analyzed using the t-test with a significance level of <0.05. Multivariate logistic regression for predicting risk factors of VTE after craniotomy/craniectomy was performed for variables chosen a priori including age, post-operative days before anticoagulant administration, female sex, trauma as an indication for surgery, tumor as an indication for surgery, presence of PICC line, and anticoagulant administration.

Since not all patients received anticoagulant prophylaxis, the average of “post-operative days before anticoagulant administration” (2.738) was used to fill in the blanks. Additionally, the variable presence of anticoagulant administration was used to determine if significance could be attributed to this estimation. A patient was considered to have an anticoagulant administered if therapy was initiated within 30 days after craniotomy/craniectomy. The Institutional Review Board of Arrowhead Regional Medical Center approved this study and did not require informed consent for the use of deidentified data.

## Results

This study included 197 patients (39.6% female; mean age 53.8±15.7 years) with the following indications for craniotomy/craniectomy: tumor (43.1%), trauma (22.3%), and others (34.5%) (Table 1). Our analysis also included an “others” category, which included cranioplasty that occurs months after severe head trauma and ventriculoperitoneal shunt placements.

**Table 1:** Patient demographics such as age, gender, and indication for surgery.

(n = 197)	n	%
Age		
18-44	52	26.4
45-63	86	43.7
64-74	42	21.3
75-99	17	8.6
Gender		
Male	119	60.4
Female	78	39.6
Surgery indication		
Tumor	85	43.1
Meningioma	12	14.1
Glioblastoma	6	7.1
Pituitary adenoma	9	10.6
Others	58	68.2
Trauma	44	22.3
Epidural hematoma	5	11.4
Subdural hematoma	26	59.1
Intraparenchymal hematoma	3	6.8
Intraventricular hematoma	1	2.3
Unspecified intracranial hematoma	5	11.4
Depressed skull fracture	3	6.8
Skull biopsy	1	2.3
Abscess evacuation	1	2.3
Autologous cranioplasty	1	2.3
Others	68	34.5

There were 24 cases of VTE (12.2%), 9 cases of DVT (4.6%), and 19 cases of SVT (9.6%). Four patients developed both DVT and SVT and were included in each category. VTE data was stratified by DVT and SVT and compared to patients without VTE (Table 2).

**Table 2:** Incidence of different types of VTE based on studied risk factors. ^a^Four patients developed both DVT and SVT and were included in DVT, SVT, and VTE columns. DVT: deep vein thrombosis; SVT: superficial vein thrombosis; VTE: venous thromboembolism

Characteristic	DVT (n=9)	SVT (n=19)	VTE (n=24)^a^	No VTE (n=173)	p Value
Age	43.8 ± 10.7	56.6 ± 19.3	55.1 ± 17.7	53.7 ± 15.4	0.715
Gender					
Male	8 (89.9%)	13 (68.4%)	17 (70.8%)	102 (59.0%)	0.265
Female	1 (11.1%)	6 (31.6%)	7 (29.2%)	71 (41.0%)	0.265
Laterality					
Right	3 (33.3%)	6 (31.6%)	6 (25.0%)	NA	
Left	4 (44.4%)	3 (15.8%)	7 (29.2%)	NA	
Bilateral	2 (11.1%)	10 (52.6%)	11 (45.8%)	NA	
PICC line	2 (22.2%)	2 (10.5%)	4 (16.7%)	12 (6.9%)	0.102
Anticoagulant administered (heparin)	8 (88.9%)	12 (63.2%)	16 (66.7%)	133 (76.9%)	0.275
Post-operative days before anticoagulant administration	4.4 ± 4.1	7.1 ± 8.7	7.2 ± 7.7	2.2 ± 3.6	0.024***
Indication for surgery					
Tumor	2 (22.2%)	3 (15.8%)	5 (20.8%)	80 (46.2%)	0.019***
Trauma	2 (22.2%)	8 (42.1%)	9 (37.5%)	35 (20.2%)	0.057
Other	5 (55.5%)	8 (42.1%)	10 (41.7%)	58 (33.5%)	0.432

Laterality was assessed, and there was no significant relationship between location and DVT or SVT (ꭓ^2 ^= 3.33, p = 0.189). In all the four patients with PICC lines who developed VTE, VTE developed contralateral to the PICC line placement. Anticoagulant was administered to 66.7% of VTE patients compared to 73.9% of patients who did not develop VTE. Among patients who received anticoagulants, 142 patients received heparin (95.3%), five received lovenox (3.4%), and two received warfarin (1.3%). Anticoagulants were administered 7.2±7.7 days after craniotomy/craniectomy among patients who developed VTE, which is a significantly greater gap compared to patients who did not develop VTE (2.2±3.6 days; p = 0.024). A significant relationship was found between tumor as an indication for craniotomy/craniectomy and whether a patient developed VTE (ꭓ^2 ^= 5.55, p = 0.019).

Table 3 shows the logistic regression model for predicting VTE and controlling for age, female sex, tumor as an indication for surgery, trauma as an indication for surgery, presence of PICC line, and anticoagulant administration.

**Table 3:** Multivariate logistic regression analysis of studied risk factors for VTE. VTE: venous thromboembolism; CI: confidence interval; PICC: peripherally inserted central catheter

	Variables	Odds ratio	95% CI	p-value
X_0_	intercept	0.073		0.010***
X_1_	Age	1.020	0.989–1.052	0.219
X_2_	Post-operative days before anticoagulant administration	1.183	1.068–1.311	0.001***
X_3_	Female	0.554	0.188–1.629	0.283
X_4_	Trauma	1.150	0.381–3.472	0.804
X_5_	Tumor	0.286	0.081–1.011	0.052
X_6_	PICC line	1.874	0.409–8.583	0.418
X_7_	Anticoagulant administered (heparin)	0.473	0.175–1.281	0.141

**Figure 1:**
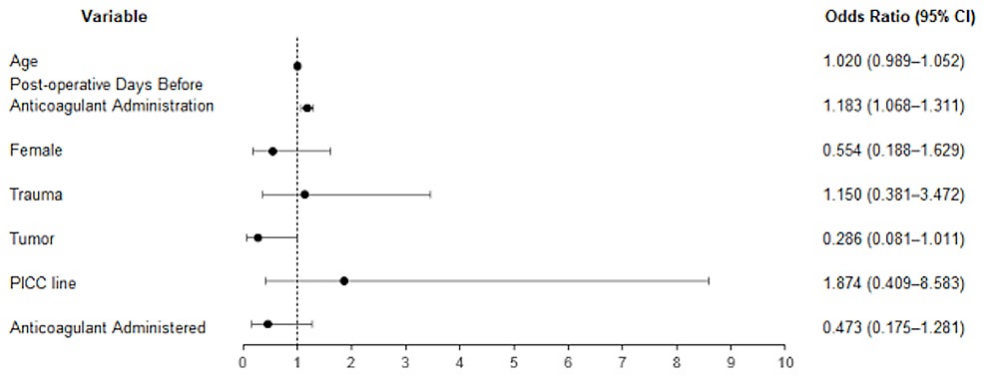
Forest plot of multivariate logistic regression output with x-axis showing odds ratio with a 95% CI. CI: confidence interval

The model found that increasing the number of days between surgery and the administration of anticoagulants significantly increased the risk of VTE incidence (odds ratio 1.183, 95% CI 1.068-1.311, p = 0.001) (Figure 1).

This model did not find the significance of the indication for craniotomy/craniectomy in predicting VTE incidence. The logistic regression equation is logit (P) = -2.614 + X_1_*0.019 + X_2_*0.168 - X_3_*0.591 + X_4_*0.140 - X_5_*1.252 + X_6_*0.628 - X_7_*0.748. This equation was statistically significant by Pearson's chi-squared test (ꭓ^2 ^= 22.592, p = 0.002).

## Discussion

This retrospective study aimed to identify the incidence and risk factors associated with DVT and SVT among post-craniotomy/craniectomy patients. The incidences of DVT, SVT, and VTE were 4.6%, 9.6%, and 12.2%, respectively. The logistic regression model did not find the indication for craniotomy/craniectomy to be significant in predicting VTE incidence, despite the relationships of TBI, brain tumors, and brain surgery with hypercoagulability proposed in the literature [6-13]. However, this model showed that increasing the number of days between surgery and the administration of anticoagulants significantly increased the risk of VTE incidence.

**Figure 2:**
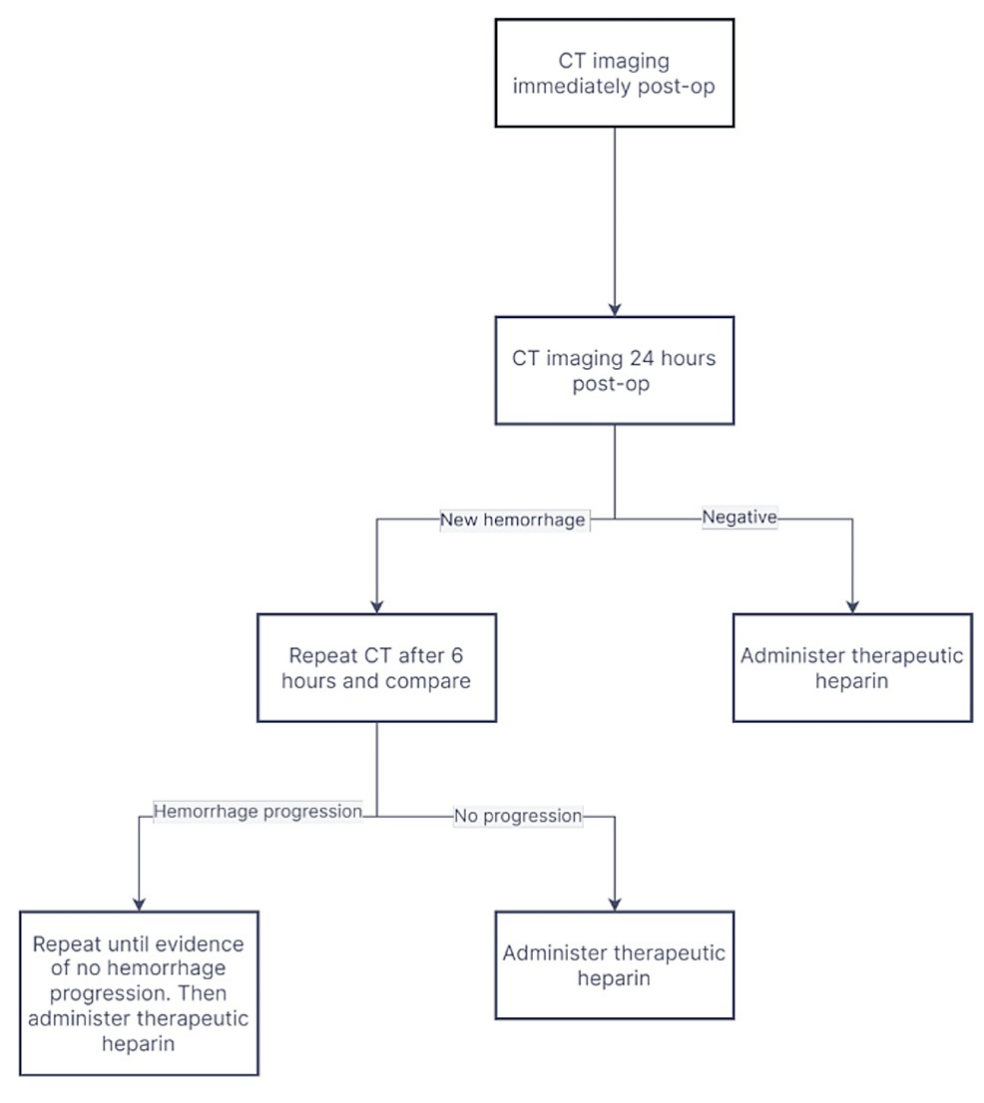
Protocol for administering anticoagulation therapy in post-craniotomy patients. CT imaging is done daily until there is evidence of no new bleeding, and subsequently, therapeutic heparin is administered.

Currently, there are no universal guidelines for the use of anticoagulants as prophylaxis in neurosurgical patients, and more generally, there is no consensus on the preferred VTE risk assessment tool [16]. The protocol at our center for administering anticoagulant therapy to post-craniotomy patients is that a pair of stable CT scans taken 24 hours apart must show no signs of new hemorrhage or hemorrhage progression (Figure 2).

This can result in the initiation of therapeutic heparin as soon as 24 hours after surgery. However, treatment approaches vary across institutions. Broadly, VTE assessment models can be classified as qualitative or quantitative, with the latter lauded for its scientifically sound individualized point scoring and criticized for its cumbersome implementation.

The classic “3 Bucket” model, which was developed at the University of California San Diego and derived from the AT8 guideline, is the most common model in the United States. This qualitative model defines three ordinal VTE risk groups. Each risk group is based on characteristics such as minor surgery (low risk) and multiple major trauma (high risk), and the groups are associated with escalating treatment protocols: no prophylaxis, unfractionated heparin or low-molecular-weight heparin (LMWH), or IPCD and LMWH [16]. Although the high incidence of VTE in neurosurgical patients has been reported in previous literature and this study, in this model, neurosurgery is not listed as a risk factor [2,16].

The Caprini risk assessment model (RAM) is an individualized quantitative VTE risk assessment model, which, until recently, has been the only externally validated point-based model. Four risk tiers are associated with the Caprini score ranges and have been derived from a checklist of items evaluated by the provider. Similar to the a priori risk factors selected in this study, the checklist includes items such as age, tumors, and PICC lines [16]. Although the Caprini model has a greater theoretical potential to prevent VTE, it is only efficacious if patient data are conscientiously analyzed and used to create real-time adjustments [17,18].

While anticoagulant therapy has the potential to decrease morbidity and mortality among neurosurgical patients, VTE prophylaxis usage should be balanced with the bleeding, discomfort, expense, and other adverse effects associated with prophylactic treatment [16]. However, it has been shown that hemorrhagic complications are low (2.2%) among patients undergoing neurosurgical resection of brain tumors when medical prophylaxis is initiated on the post-operative day [5,19]. Similarly, it has been shown that patients undergoing craniotomy/craniectomy for TBI and on antiplatelet/anticoagulation therapy have no significant difference in bleeding events [20].

Future prospective studies assessing VTE risk assessment models such as “3 Bucket” or Caprini to develop universal guidelines for administering anticoagulant therapy to post-craniotomy/craniectomy patients, including the timing of post-operative therapy initiation, could have widespread clinical implications.

A limitation of this study is the lack of standardization due to its retrospective study design. Selection bias occurred because patients who received anticoagulant therapy were at a perceived higher risk of developing a VTE than patients who did not receive anticoagulant therapy. Additionally, the timing of anticoagulant administration was not standardized; the number of post-operative days before anticoagulant administration was one day. Lastly, the anticoagulant drug administered was not standardized.

## Conclusions

Among post-craniotomy/craniectomy patients, incidences of DVT, SVT, and VTE were 4.6%, 9.6%, and 12.2%, respectively. The logistic regression model did not find the indication for craniotomy/craniectomy, including tumor and trauma, to be significant. However, the model showed that increasing the number of days between surgery and the administration of anticoagulants significantly increased the risk of VTE incidence. Future prospective studies should assess VTE risk assessment models such as “3 Bucket” or Caprini to develop universal guidelines for administering anticoagulant therapy to post-craniotomy/craniectomy patients with a consideration of the timing of post-operative therapy initiation.
